# Identification of Potential Long Non-Coding RNA Candidates that Contribute to Triple-Negative Breast Cancer in Humans through Computational Approach

**DOI:** 10.3390/ijms222212359

**Published:** 2021-11-16

**Authors:** Md. Motiar Rahman, Md. Tofazzal Hossain, Md. Selim Reza, Yin Peng, Shengzhong Feng, Yanjie Wei

**Affiliations:** 1 Department of Biochemistry and Molecular Biology, University of Rajshahi, Rajshahi 6205, Bangladesh; 2 Department of Chemistry, Binghamton University, State University of New York, Vestal, New York, NY 13902, USA; 3 University of Chinese Academy of Sciences, No.19(A) Yuquan Road, Shijingshan District, Beijing 100049, China; tofazzal.stat@gmail.com (T.H.); selim@siat.ac.cn (S.R.); 4 Joint Engineering Research Center for Health Big Data Intelligent Analysis Technology, Shenzhen Institutes of Advanced Technology, Chinese Academy of Sciences, Shenzhen 518055, China; sz.feng@siat.ac.cn; 5 Department of Statistics, Bangabandhu Sheikh Mujibur Rahaman Science and Technology University, Gopalganj 8100, Bangladesh; 6 Department of Pathology, The Shenzhen University School of Medicine, Shenzhen 518060, China; ypeng@szu.edu.cn

**Keywords:** long non-coding RNA, lncRNA biomarker, triple-negative breast cancer

## Abstract

Breast cancer (BC) is the most frequent malignancy identified in adult females, resulting in enormous financial losses worldwide. Owing to the heterogeneity as well as various molecular subtypes, the molecular pathways underlying carcinogenesis in various forms of BC are distinct. Therefore, the advancement of alternative therapy is required to combat the ailment. Recent analyses propose that long non-coding RNAs (lncRNAs) perform an essential function in controlling immune response, and therefore, may provide essential information about the disorder. However, their function in patients with triple-negative BC (TNBC) has not been explored in detail. Here, we analyzed the changes in the genomic expression of messenger RNA (mRNA) and lncRNA in standard control in response to cancer metastasis using publicly available single-cell RNA-Seq data. We identified a total of 197 potentially novel lncRNAs in TNBC patients of which 86 were differentially upregulated and 111 were differentially downregulated. In addition, among the 909 candidate lncRNA transcripts, 19 were significantly differentially expressed (DE) of which three were upregulated and 16 were downregulated. On the other hand, 1901 mRNA transcripts were significantly DE of which 1110 were upregulated and 791 were downregulated by TNBCs subtypes. The Gene Ontology (GO) analyses showed that some of the host genes were enriched in various biological, molecular, and cellular functions. The Kyoto encyclopedia of genes and genomes (KEGG) pathway analysis showed that some of the genes were involved in only one pathway of prostate cancer. The lncRNA-miRNA-gene network analysis showed that the lncRNAs TCONS_00076394 and TCONS_00051377 interacted with breast cancer-related micro RNAs (miRNAs) and the host genes of these lncRNAs were also functionally related to breast cancer. Thus, this study provides novel lncRNAs as potential biomarkers for the therapeutic intervention of this cancer subtype.

## 1. Introduction

BC is regarded as the most frequently observed cancer in females throughout the world [1,2,3], and distance metastasis is the foremost cause of lower survival rate [4,5]. TNBC is a subgroup of BCs that lack the expression of estrogen receptor (ER), and progesterone receptor (PR), as well as human epidermal growth factor receptor 2 (HER-2) [6,7]. In addition, the possibility of distant metastasis along with the recurrence in patients with TNBC is relatively higher when compared to normal patients [8]. So the management strategies of TNBC are somewhat difficult due to the comparatively aggressive biological features and deficiency of distinct targeted medication [9]. Therefore, there is a need to acquire a good understanding of the mechanisms and regulations of tumorigenesis in TNBC cells as well as the elucidation of efficient biomarkers for the prognosis and diagnosis of TNBC patients. To this line, identification of the novel metastasis-related molecular pathway is of great importance to developing the consequence of TNBC treatments.

Recently, it was revealed that long non-coding RNAs (lncRNAs) have close interactions with BC metastasis [10,11,12,13]. LncRNAs are a class of non-coding RNAs with a length of >200 nucleotides, which are linked with many biological processes, such as cancer cell invasion, proliferation, apoptosis, differentiation, development, and metastasis [14,15]. So far, lncRNAs have been exhibited to play auxiliary roles either to tumorigenesis or to tumor suppression. Specifically, in BC, increasing data has strengthened the hypothesis that lncRNAs exert an essential function in controlling BC metastasis [16,17,18]. Many lncRNAs were underregulated abnormally in a variety of BC cells [3,19,20,21], ovarian cancer [22], hepatocellular carcinoma [23], and many others [14,24]. Numerous studies have suggested that various lncRNAs, including HOTAIR, BCAR4, and linc-ROR, were upregulated and stimulated BC invasion as well as metastasis. HOTAIR upregulation was shown to be strongly related to lymph node metastasis in patients with TNBCs [25], despite the fact of how it controls lymph node metastasis, as well as BC lung metastasis, are not well understood. Therefore, it demands extensive studies about the roles of lncRNA in TNBCs.

Latest studies have discovered that lncRNAs play a crucial role in the tumorigenesis and progression in TNBC. SOX21-AS1, HOST2, HUMT, XIST, FAM83H-AS1, and LINC00173, for example, were reported as potential candidates in the initiation and development of TNBC [26,27,28,29,30]. Several other studies have identified AFAP1-AS1, MALAT1, NRON, and RMST as significant regulators of TNBC-mediated cell proliferation, migration, metastasis, and tumorigenicity [31,32,33,34]. Similarly, lncRNA MIR100HG and LINC01638 regulate cell proliferation [35,36], MTDH enhances breast tumorigenicity [37] and SNHG12 regulates cell proliferation and migration in such breast cancer types [38].

It was exhibited that lncRNAs may function as agonists or antagonists of transcription, protein scaffolds, miRNA sponges, or antisense RNA [39]. They are recognized to have lower evolutionary conservation and show lower cellular concentration compared to protein-coding transcripts, but possess a higher degree of tissue specificity [40]. Nevertheless, despite having an outstanding role in the regulation of gene expression, lncRNAs were poorly identified and annotated in TNBC patients [41]. The objective of this study is to deepen the knowledge about the crucial interplay between lncRNAs expression and TNBCs and provide novel insight into the regulatory function of lncRNAs in humans during TNBCs. To this end, we used the bioinformatics pipeline to identify the lncRNAs expression profiles and their putative role in TNBCs patients using publicly available single-cell RNA-seq data.

## 2. Results

### 2.1. Identification of lncRNA Transcripts

We analyzed scRNASeq data of 60 TNBC samples collected from six patients (10 samples per patient) and 20 normal samples to identify potential lncRNAs responsible for TNBCs. A total of 83,401 transcripts from 80 samples were identified. Firstly, 10,385 transcripts with a class code “j” having unknown annotations were set for selecting the putative lncRNAs. Then, a filtering strategy was adopted to filter out low-quality assemblies such as those having <200 bp for more than one exon transcript and <500 bp for single-exon transcripts. The minimum length of the transcripts with a class “j” was 255 and there were no single-exon transcripts. Therefore, all the transcripts with a class code “j” passed the filtering step. The protein-coding ability of the 10,385 transcripts was assessed by the coding potential calculator 2 (CPC2). The threshold cut-off for coding probability *p* < 0.5 was used to find the non-coding candidate transcripts as the standard rule of CPC2 is to declare any transcript having a coding probability of *p* < 0.5 as “non-coding” and “coding” otherwise. Finally, a total of 909 candidate lncRNAs were detected.

### 2.2. Expression Profiles of lncRNAs and mRNAs

**Figure 1:**
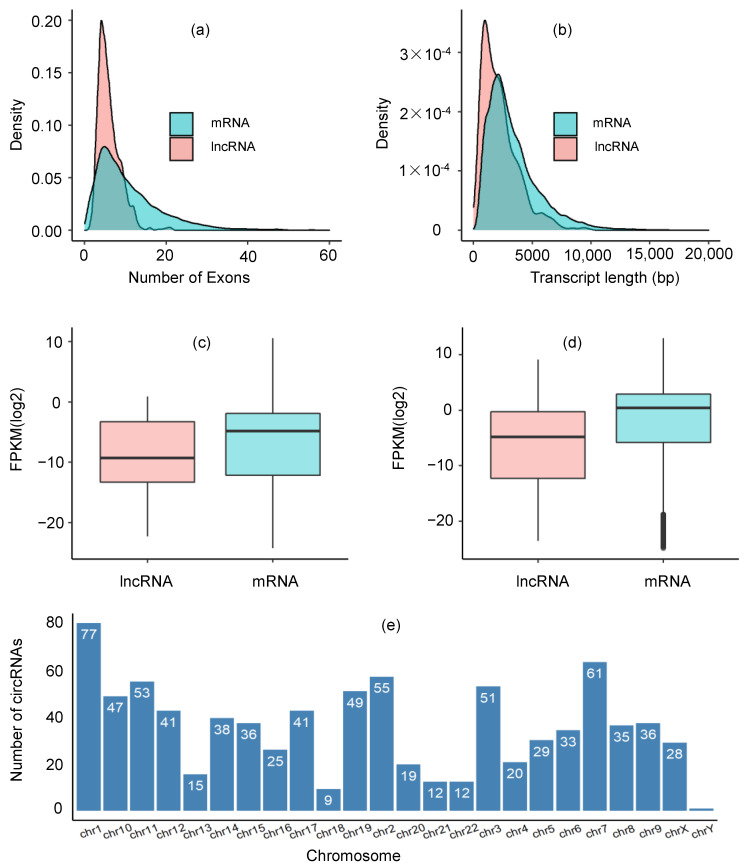
Expression profile of candidate lncRNA transcripts. Density plot for the number of exons in mRNA and lncRNA transcripts (**a**), the length of mRNA and lncRNA transcripts (**b**), expression levels (FPKM) of mRNA and lncRNA transcripts in normal samples (**c**), expression levels (FPKM) of mRNA and lncRNA transcripts in TNBC patients (**d**) and chromosome distribution of the candidate lncRNAs (**e**).

In comparison to other RNAs, lncRNAs have been demonstrated to exhibit distinct characteristics, such as lower levels of cellular concentration, fewer exons, and shorter length than protein-coding genes [41]. In this study, we compared the number of exons and their length, chromosome distribution, and expression between lncRNAs and mRNAs in our dataset. It was shown that the number of mRNA exons (median 9) was greater than that of lncRNAs exons (median 5), and the number was statistically significant which was determined by a two-tailed Mann–Whitney U-test (*p* = 2.20 × 10^−16^). We also observed, in lncRNAs, about 66% of the transcripts having ≤ 6 exons and 34% of the transcripts having ≤ 4 exons. The length of lncRNAs transcript (median 1899) was smaller than that of mRNAs (median 2780) with *p* = 2.20 × 10^−16^ determined by a two-tailed Mann–Whitney U-test. Similarly, the level of lncRNAs expression was smaller than that of mRNAs with mean FPKM values 6.53 and 17.13, respectively. The Student’s *t*-test provided evidence that the difference in expression levels between lncRNAs (mean FPKM 6.53) and mRNA (mean FPKM 17.13) was statistically significant with *p* = 0.01625. The distribution of lncRNAs across chromosomes was heterogeneous, and most of the lncRNAs exist in chr1 (77/909, 8.47%). Compared to other chromosomes, the chr1, chr2, chr3, chr7, chr10, chr11, and chr19 possessed more lncRNAs, which accounted for more than 5% of total potential lncRNAs in each chromosome and the average number of lncRNAs per chromosome was 34. The expression profiles of lncRNAs and mRNAs have been shown in Figure 1.

### 2.3. Differential Expression Analysis of lncRNAs and mRNAs

**Figure 2:**
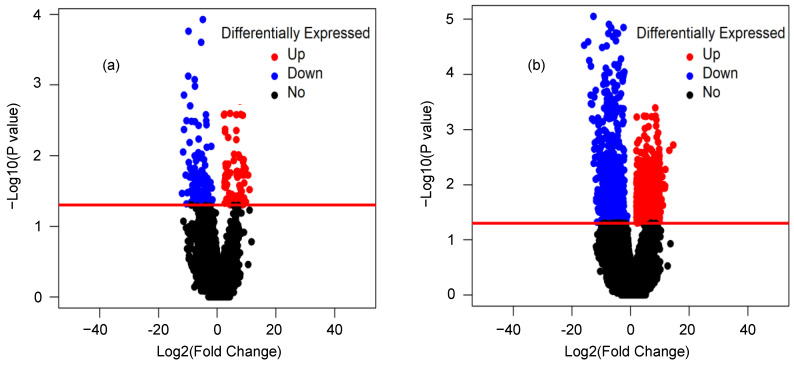
Volcano plot for the output of DESeq. LncRNA transcripts (**a**) and mRNA transcripts (**b**).

**Figure 3:**
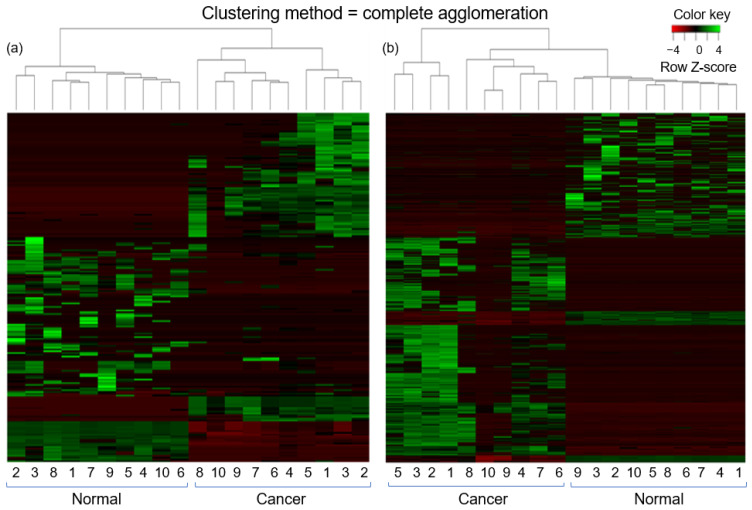
Heatmap of the DE transcripts. (**a**) lncRNA (**b**) mRNA.

We next identified the differentially expressed (DE) lncRNAs and mRNAs between two groups (normal vs. cancer). The R package DESeq was applied for this purpose. The volcano plot was used to find out the DE lncRNAs and mRNAs with the thresholds *p*-value < 0.05 and foldchange > 2.0 (Figure 2). Out of a total of 55,728 mRNA transcripts, 1901 mRNA transcripts were significantly DE. Among the significant DE mRNA transcripts, 1110 were upregulated and 791 were downregulated. On the other hand, out of a total of 15,308 lncRNA transcripts, 197 transcripts were significantly DE of which 86 were upregulated and 111 were downregulated. Similarly, among the 909 candidate lncRNA transcripts, 19 were significantly DE of which three were upregulated and 16 were downregulated. Table 1 and Table 2 represented the top 20 DE (10 upregulated and 10 downregulated) transcripts for lncRNA and mRNA, respectively. In addition, the top 10 DE transcripts (three upregulated and seven downregulated) for candidate lncRNAs were given in Table 3. The expression patterns of lncRNA and mRNA transcripts were shown in the heatmap (Figure 3). The dendrograms of the two groups (tumor vs. normal) were clearly distinguishable. For the heatmap, 10 normal and 10 cancer samples were generated by averaging 20 and 60 samples, respectively.

### 2.4. Analysis of lncRNA-miRNA-Gene Interaction

**Figure 4:**
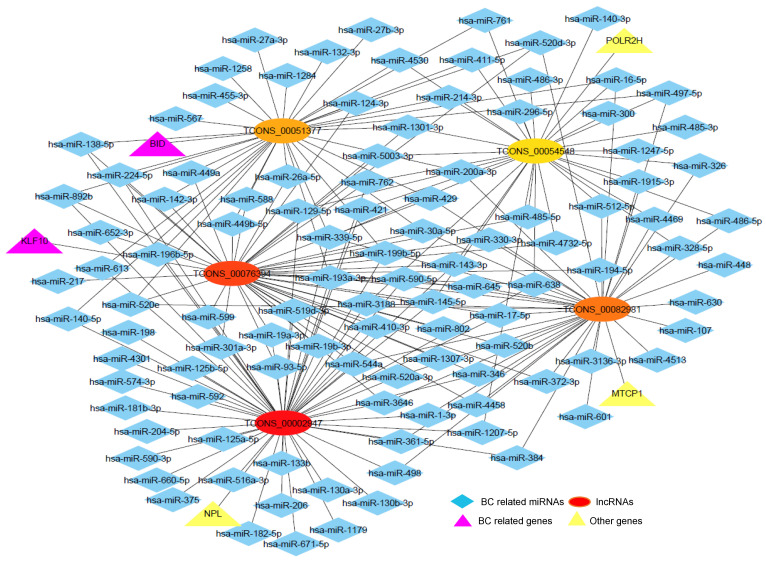
LncRNA-miRNA-gene interaction network. The lncRNAs are the top 5 hub lncRNAs where the deepness of the red color indicates a higher degree of lncRNAs. All the miRNAs are related with BC. The genes are the host genes of the lncRNAs.

We next studied the interaction between lncRNA and miRNA using the prediction tool miRanda. For this purpose, the miRNA sequences were downloaded from the mirbase database. A total of 6011 interactions between 10 lncRNA and 2175 miRNA were observed. From the miRCancer database, cancer-related miRNAs were downloaded, and 654 BC-related miRNA candidates were identified. There were 118 miRNAs common between 654 BC-related miRNAs and 2175 miRNAs that were observed in the interaction network. The interactions for these 118 miRNAs were extracted from the total 6011 interactions, and a total of 315 interactions were observed. Then, a lncRNA-miRNA-gene network was constructed using Cytoscape. The genes in the network were the host genes of the lncRNAs. From this network, a subnetwork was constructed with the top five hub lncRNAs (Figure 4). The deepness of the red color indicates a higher degree of lncRNA. Here, the degree of a lncRNA means the number of miRNAs and genes connected to that lncRNA. From the network, it was observed that two genes, BID and KLF10 (pink colored), were functionally related to BC. Two lncRNAs, TCONS_00076394 and TCONS_00051377, were interacted with BC-related miRNAs and their host genes were also functionally related to BC. This indicated that these two lncRNAs might be important biomarkers for TNBC.

### 2.5. GO Term and KEGG Pathway Enrichment Analysis

We downloaded the PPI network from the STRING database for the 197 host genes of the DE lncRNA transcripts and found that 35 genes participated in the PPI network. We then performed the GO and KEGG pathways analyses for these genes. The GO term biological process analysis showed that some of the host genes were involved with the positive regulation of cell division, neutrophil chemotaxis, creatine metabolism, positive regulation of protein phosphorylation, defense response to protozoan, and negative regulation of cell proliferation. The GO term molecular function analysis showed that host genes were enriched in transferase activity, transferring phosphorus-containing groups, kinase activity, creatine kinase activity, and growth factor activity. The GO term cellular component analysis showed that the host genes were enriched in the mitochondrial inner membrane. The KEGG pathway analysis showed that some of the genes were involved in the prostate cancer pathway. The significant GO term and pathways were shown in Table 4.

## 3. Discussion

TNBC is well-known as a major problem affecting the health of females worldwide and its underlying mechanism has not been revealed properly. Due to the outstanding heterogeneity of BCs, it is crucial not only to categorize them based on morphologic and clinical features but also to examine intrinsic molecular signatures. Compared to other BCs, TNBC is regarded as high malignancy, young-onset, easy recurrence, and low survival rates [42,43]. Since TNBC is deficient in ER, PR and HER2 receptors, this type of cancer has no specific targets of hormone therapy as well as a targeted treatment. In this regard, it is essential to detect alternative molecular targets for TNBC treatment. To this line, we aimed to investigate potential lncRNAs interrelated with TNBC.

In this study, single-cell RNA-Seq data were used to identify the changes in genomic expression profiles of lncRNA as well as mRNA in patients with TNBC. A comprehensive bioinformatics pipeline was adopted from the previous literature [44,45,46] for the detection of novel lncRNAs in TNBC patients. The potential biological functions of the newly identified lncRNA candidates were inferred by identifying the functional importance of adjacent protein-coding genes along with their co-expression profiles. This study provides novel statistics about the early immunity against TNBC and figures out the significance of regulatory RNAs.

Here, a total of 83,401 transcripts of which 10,385 transcripts with a class code “j” having unknown annotation were applied for the detection of the putative lncRNAs. After the filtration process, a total of 909 candidate lncRNAs were identified. Out of 909 candidate lncRNA transcripts, 19 were significantly DE of which three were upregulated and 16 were downregulated.

Previous studies have explored the particular role of lncRNAs in TNBC. *MALAT1*, for instance, has been reported as a promoter of proliferation and invasion through microRNA-129-5p [32]. The overexpression of lncRNA *MIR100HG* was related to poor prognosis while downregulation of MIR100HG significantly suppressed TNBC-induced cell proliferation, and reduced tumor growth [35]. Our identified lncRNAs candidates showed differential expression in normal vs. tumor groups. In addition, owing to the inherent regulatory nature, lncRNAs may have a greater biological impact compared to a single gene. Even the candidate lncRNAs might be associated with the TNBC by sponging miRNAs. As we identified the candidate lncRNAs through the computational approach, knowing more specific roles of the candidate lncRNAs through the experimental procedure is beyond the scope of this study. Further experiments are needed to explore more specific roles of the identified candidates.

LncRNAs have been shown to affect the expression, or function of their host gene [47]. We predicted the function of lncRNAs by GO analysis for the host genes of the differentially expressed lncRNAs. GO term analyses showed that several host genes functioned in some important biological processes, molecular mechanisms and cellular components. The KEGG pathway analysis showed that the host genes were involved in the prostate cancer pathway (Table 4).

The most important property of lncRNA is known to have a function as miRNA sponges [48]. As mRNAs play a crucial role in cancer progression, we performed lncRNA-miRNA–gene interaction network analysis to explore the relationship between the DE lncRNAs and miRNAs. From the network analysis, it was found that the five hub lncRNAs interacted with the BC-related miRNAs.

The host gene of the lncRNAs was also related functionally to BC. Two host genes BID and KLF10 were related to BC. BID was found to be a potential target in the therapeutic strategies for BC [49]. KLF10 was an anti-metastasis gene that significantly prevents BC cell invasion [50]. KLF10 was involved in cell proliferation and apoptosis, and its levels were inversely associated with BC stages, implying that it had a tumor suppressor function [51]. The host genes BID and KLF10 were the important biomarkers for BC. Similarly, the lncRNAs TCONS_00076394 and TCONS_00051377 were interacted with BC-related miRNAs. These two lncRNAs were unannotated which meant that their function was unknown. As the function of lncRNAs can be predicted based on the function of their interacted miRNAs and host genes, TCONS_00076394 and TCONS_00051377, might be the potential biomarkers for BC. However, there are some limitations in this study. Firstly, the datasets that had been availed here to detect the DE candidate lncRNAs and mRNAs were adopted from a public resource as well as the fact that they were not experimentally validated. Secondly, owing to the lower sample size, pairwise comparisons of some parameters were not statistically significant enough. So that further experiments might be effective to validate our findings.

## 4. Materials and Methods

### 4.1. Data Collection

The scRNA-seq FastQ data of six patients with TNBC (PT039, PT058, PT081, PT084, PT089, and PT126) were downloaded from the public resources Sequence Read Archive (SRA) database (GEO: GSE118390) [52]. In addition, 20 normal breast epithelial cells were also downloaded, which acted as reference cells to compare differential lncRNAs expression between normal and diseases states [53]. In this study, a total of 60 SRR files of six TNBC patients (10 from each) were included.

### 4.2. Quality Control, Mapping, and Transcript Assembly

The first step of putative RNA analysis involves sequence alignment and transcript assembly. FastQC was applied to check data quality and only good quality data were included for analyses. Sequence alignment was performed against the reference human genome (hg38) using STAR (Version STAR-2.7.9a) to search match reads and to merge duplicated and low-quality reads [54]. These data were subsequently served as input for Cufflinks for transcript assembly [55]. Gene transfer files (gtf) generated by Cufflinks were merged by Cuffmerge. Cufflinks gffread was applied to fetch fasta files from merged gtf files. To achieve the overall assembly quality, the human genome along with the annotation files were provided as inputs to Cuffmerge.

### 4.3. Transcripts Filtration

Fasta files containing less than 200 nucleotides were discarded to find out potential lncRNAs from the transcripts produced by Cufflinks. It has been demonstrated that several lncRNAs contain only single-exon transcripts [44,56,57]. These single-exon transcripts; however, are often removed since they can lead to generating background noise as a result of inaccurate transcript assembly, experimental artifacts, or genomic contamination during sequence library preparation. To overcome this problem, Sun et al. [58] employed a stringent size threshold of 1000 bp for identifying single-exon transcripts. Later, Bush et al. [59] used a size threshold of 500 bp that we applied in this assay, considering the shortcomings and the point that the mean length of single-exon transcript in humans is reported to be <300 bp [60]. The full-length sequences for all transcripts (obtained from Cufflinks gffread) were considered as the reference genome and the fastq reads were mapped using the bowtie2 aligner. Then, count data was generated using bedtools multiBamCov with the output of bowtie2 (converted to bam, sorted, and indexed). The count data was used to identify differentially expressed (DE) transcripts by the R package DESeq. Foldchange > 2 and *p*-value < 0.05 were considered as the cut-off for defining significant DE transcripts.

### 4.4. Assessment of the Coding Potential

**Figure 5:**
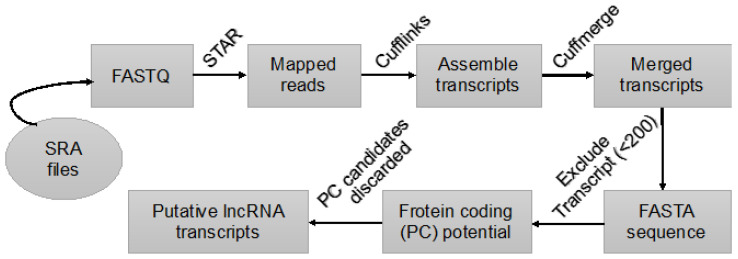
In silico identification method of lncRNAs from scRNA-seq databases.

In this study, protein-coding potential (PCP) of shortlisted lncRNAs were checked using a coding-potential-calculator 2 (CPC2) [61]. CPC2 uses sequence hallmark to differentiate between coding and non-coding RNAs, hence, DNA sequences of the transcripts were extracted applying Cufflink’s gffread [59]. A cut-off coding probability of *p* < 0.5 for CPC2 was chosen to treat a transcript as non-coding and to decrease false-positive identification [44]. The detailed pipeline of identifying lncRNA candidates is depicted in Figure 5.

### 4.5. Differential Expression Analysis

Differential expression analysis was done using DESeq [62]. The count data (obtained from multiBamCov-bedtools 2.30.0) was used to identify differentially expressed (DE) transcripts between the normal and TNBC group. Foldchange (>2) and *p*-value (<0.05) were considered as the cut-off for defining significant DE transcripts. A heatmap was used to differentiate the expression patterns between normal and cancer groups.

### 4.6. GO and KEGG Enrichment Analysis

After finding the DE lncRNAs, the host genes of the DE lncRNA transcripts were extracted. The host genes of DE lncRNAs were mapped to Search Tool for the Retrieval of Interacting Genes (STRING v11.5; http://string-db.org/cgi/input.pl, accessed on 3 August 2021), and the protein–protein interaction (PPI) among the genes was obtained. Then, a PPI network was constructed using the software Cytoscape. From the network, the top hub genes were selected for GO term and KEGG pathway enrichment analyses. With the selected hub genes, Gene Ontology (GO) term and KEGG pathway enrichment analyses were performed using DAVID to know the function of the lncRNAs. The threshold *p*-value < 0.05 was used for the significance of the enrichment analysis.

### 4.7. LncRNA–miRNA-Gene Interaction Network Analysis

The DE lncRNAs were sorted based on *p*-value and fold change. Then, the top 10 DE lncRNAs (three upregulated and seven downregulated) were selected for miRNA interaction analysis. The lncRNA-miRNA interaction was predicted using miRanda software. The miRNA sequences were downloaded from mirbase. From the miRCancer database, the miRNAs related to BC were also downloaded. From the lncRNA–miRNA interaction network, a sub-network was constructed keeping only the BC-related miRNAs. Finally, the lncRNA-miRNA-gene network was constructed using Cytoscape where the genes were the host genes of the DE lncRNAs.

## 5. Conclusions

In this study, we investigated potential lncRNAs interrelated with TNBC using single-cell RNA-Seq data and found two lncRNAs TCONS_00076394 and TCONS_00051377 as potential biomarkers for TNBC. These two lncRNAs were differentially expressed between cancer and normal groups, interacted with breast cancer-related miRNAs, and their host genes were also functionally related to breast cancer. Thus, we can conclude that these two lncRNAs TCONS_00076394 and TCONS_00051377 might be considered as potential biomarkers for TNBC. Further experiments are required to know the specific function of these lncRNAs in TNBC.

## Figures and Tables

**Table 1:** Top 20 (10 upregulated and 10 downregulated) DE mRNAs. DE mRNAs were sorted based on *p*-value and fold change.

mRNA ID	Location	No. of Exons	Length	Base Mean	LFC	*p*-Value	Gene
TCONS_00027847	chr16:72054592-72061056	7	1448	3419.94	Inf	0.00041	HP
TCONS_00062848	chr5:150401637-150412936	9	1681	2201.54	Inf	0.00058	CD74
TCONS_00027845	chr16:72054592-72061056	5	1271	2210.50	Inf	0.00058	HP
TCONS_00062849	chr5:150401637-150412936	8	1489	2166.03	Inf	0.00058	CD74
TCONS_00037794	chr19:6677835-6720682	41	5132	2131.98	Inf	0.00059	C3
TCONS_00027846	chr16:72054592-72061056	5	1271	2125.11	Inf	0.00060	HP
TCONS_00030302	chr17:34255277-34257201	3	747	1907.13	Inf	0.00067	CCL2
TCONS_00008098	chr10:58513144-58831437	21	5471	1317.61	Inf	0.00087	BICC1
TCONS_00062847	chr5:150401637-150412936	6	1305	1274.15	Inf	0.00089	CD74
TCONS_00005374	chr1:89052304-89065360	11	3050	1164.10	Inf	0.00096	GBP1
TCONS_00065563	chr6:27866792-27867581	1	790	301.37	−12.6	8.98 × 10^−06^	HIST1H1B
TCONS_00028779	chr16:30104810-30113557	10	1098	105.09	−Inf	1.25 × 10^−05^	GDPD3
TCONS_00010969	chr10:128096661-128126204	14	11,415	114.64	−Inf	1.44 × 10^−05^	MKI67
TCONS_00010970	chr10:128096661-128126204	15	12,495	119.33	−Inf	1.45 × 10^−05^	MKI67
TCONS_00012090	chr11:62270155-62273157	3	506	117.08	−Inf	1.80 × 10^−05^	SCGB2A2
TCONS_00031424	chr17:79778178-79787650	5	4265	42.19	−Inf	1.84 × 10^−05^	CBX2
TCONS_00082965	chrX:154651972-154653579	3	764	24.78	−Inf	1.85 × 10^−05^	CTAG2
TCONS_00054384	chr3:170037947-170085395	4	2013	18.08	−Inf	2.11 × 10^−05^	GPR160
TCONS_00082964	chrX:154651972-154653579	2	993	20.37	−Inf	2.50 × 10^−05^	CTAG2
TCONS_00018204	chr12:52806543-52814116	9	2147	45.76	−14.4	2.61 × 10^−05^	KRT4

LFC = log2FoldChange and Inf = Infinite.

**Table 2:** Top 20 (10 upregulated and 10 downregulated) DE lncRNAs. DE lncRNAs were sorted based on *p*-value.

lncRNA ID	Location	No. of Exons	Length	Base Mean	LFC	*p*-Value	Gene
TCONS_00057817	chr4:78645994-78684501	4	4214	362.9557	Inf	0.00254	LINC01094
TCONS_00057819	chr4:78645994-78684501	3	4120	352.7188	Inf	0.00260	LINC01094
TCONS_00057820	chr4:78645994-78684501	3	4105	345.6054	Inf	0.00263	LINC01094
TCONS_00057818	chr4:78645994-78684501	4	4192	339.0413	Inf	0.00266	LINC01094
TCONS_00057821	chr4:78645994-78684501	3	4066	338.1632	Inf	0.00268	LINC01094
TCONS_00057816	chr4:78645994-78684501	5	4286	331.5815	Inf	0.00270	LINC01094
TCONS_00006590	chr1:169690665-169708856	7	2206	176.9738	Inf	0.00426	SELL
TCONS_00062278	chr5:91368632-91383373	8	4332	208.4584	Inf	0.00443	ARRDC3
TCONS_00062275	chr5:91368632-91380297	8	4621	200.0444	Inf	0.00463	ARRDC3
TCONS_00009315	chr10:17214239-17229985	3	1875	121.2627	Inf	0.00557	VIM-AS1
TCONS_00082872	chrX:152708261-152714549	5	825	2.665319	−Inf	0.00012	CSAG3
TCONS_00081533	chrX:152753921-152760222	5	825	2.704539	−Inf	0.00018	CSAG3
TCONS_00035347	chr19:751113-764319	4	1048	12.04587	−Inf	0.00025	MISP
TCONS_00017579	chr12:6848808-6851930	5	1615	5.361052	−Inf	0.00076	CDCA3
TCONS_00017578	chr12:6848808-6851930	6	1792	5.745075	−Inf	0.00085	CDCA3
TCONS_00012837	chr11:111912736-111926871	5	1527	30.57432	−Inf	0.00105	HSPB2-C11orf52
TCONS_00024012	chr15:43593834-43599406	9	2842	10.85619	−11.3	0.00140	CKMT1B
TCONS_00081534	chrX:152753921-152760222	5	789	0.786254	−Inf	0.00198	CSAG3
TCONS_00082873	chrX:152708261-152714549	5	789	0.968629	−Inf	0.00266	CSAG3
TCONS_00082981	chrX:155061625-155071272	6	2755	2.456605	−Inf	0.00322	MTCP1

LFC = log2FoldChange and Inf = Infinite.

**Table 3:** Top 10 (3 upregulated and 7 downregulated) DE candidate lncRNAs.

lncRNA ID	Location	No. of Exons	Length	Base Mean	LFC	*p*-Value	Gene
TCONS_00076394	chr8:102648777-102655902	4	2309	36.34	Inf	0.0146	KLF10
TCONS_00002947	chr1:182789449-182830384	11	2728	32.38	Inf	0.0391	NPL
TCONS_00051377	chr22:17734140-17774665	5	1901	9.47	Inf	0.0434	BID
TCONS_00082872	chrX:152708261-152714549	5	825	2.67	−Inf	0.0001	CSAG3
TCONS_00081533	chrX:152753921-152760222	5	825	2.70	−Inf	0.0002	CSAG3
TCONS_00082981	chrX:155061625-155071272	6	2755	2.46	−Inf	0.0032	MTCP1
TCONS_00026822	chr16:4788397-4796491	6	883	22.91	−7.6	0.0033	SMIM22
TCONS_00049160	chr20:47298126-47356889	5	1346	2.07	−3.5	0.0076	ZMYND8
TCONS_00054549	chr3:184361714-184368595	5	1723	4.02	−6.8	0.0220	POLR2H
TCONS_00054548	chr3:184361714-184368595	6	1871	4.60	−5.9	0.0233	POLR2H

LFC = log2FoldChange and Inf = Infinite.

**Table 4:** Significant GO terms and pathways of 35 genes from the PPI networks of host genes of the DE lncRNAs.

GO/Pathway ID	GO/Pathway Name	No. of Genes	*p*-Value
Biological Process			
GO:0051781	Positive regulation of cell division	3	0.0036
GO:0030593	Neutrophil chemotaxis	3	0.0070
GO:0006600	Creatine metabolic process	2	0.0208
GO:0001934	Positive regulation of protein phosphorylation	3	0.0243
GO:0042832	Defense response to protozoan	2	0.0356
GO:0008285	Negative regulation of cell proliferation	4	0.0390
Cellular Component			
GO:0005743	Mitochondrial inner membrane	4	0.0449
Molecular Function			
GO:0016772	Transferase activity, transferring phosphorus-containing groups	2	0.0094
GO:0016301	Kinase activity	4	0.0105
GO:0004111	Creatine kinase activity	2	0.0113
GO:0008083	Growth factor activity	3	0.0376
KEGG pathway			
hsa05215	Prostate cancer	3	0.0265
